# Antiviral Use Among Children Hospitalized With Laboratory-Confirmed Influenza Illness: A Prospective, Multicenter Surveillance Study

**DOI:** 10.1093/cid/ciae573

**Published:** 2024-12-17

**Authors:** James W Antoon, Justin Z Amarin, Olla Hamdan, Tess Stopczynski, Laura S Stewart, Marian G Michaels, John V Williams, Eileen J Klein, Janet A Englund, Geoffrey A Weinberg, Peter G Szilagyi, Jennifer E Schuster, Rangaraj Selvarangan, Christopher J Harrison, Julie A Boom, Leila C Sahni, Flor M Muñoz, Mary Allen Staat, Elizabeth P Schlaudecker, James D Chappell, Benjamin R Clopper, Heidi L Moline, Angela P Campbell, Andrew J Spieker, Samantha M Olson, Natasha B Halasa

**Affiliations:** Vanderbilt University Medical Center, Nashville, Tennessee, USA; Vanderbilt University Medical Center, Nashville, Tennessee, USA; Epidemiology Doctoral Program, Vanderbilt University, Nashville, Tennessee, USA; Vanderbilt University Medical Center, Nashville, Tennessee, USA; Vanderbilt University Medical Center, Nashville, Tennessee, USA; Vanderbilt University Medical Center, Nashville, Tennessee, USA; UPMC Children's Hospital of Pittsburgh, Pittsburgh, Pennsylvania, USA; UPMC Children's Hospital of Pittsburgh, Pittsburgh, Pennsylvania, USA; Department of Pediatrics, University of Wisconsin School of Medicine and Public Health, Madison, Wisconsin, USA; Seattle Children's Research Institute, Seattle, Washington, USA; Seattle Children's Research Institute, Seattle, Washington, USA; University of Rochester School of Medicine and Dentistry, Rochester, New York, USA; University of Rochester School of Medicine and Dentistry, Rochester, New York, USA; UCLA Mattel Children's Hospital and Department of Pediatrics, University of California at Los Angeles, Los Angeles, California, USA; Children's Mercy Hospital and University of Missouri–Kansas City, Kansas City, Missouri, USA; Children's Mercy Hospital and University of Missouri–Kansas City, Kansas City, Missouri, USA; Children's Mercy Hospital and University of Missouri–Kansas City, Kansas City, Missouri, USA; Texas Children's Hospital and Department of Pediatrics, Baylor College of Medicine, Houston, Texas, USA; Texas Children's Hospital and Department of Pediatrics, Baylor College of Medicine, Houston, Texas, USA; Texas Children's Hospital and Department of Pediatrics, Baylor College of Medicine, Houston, Texas, USA; Cincinnati Children's Hospital Medical Center and University of Cincinnati College of Medicine, Cincinnati, Ohio, USA; Cincinnati Children's Hospital Medical Center and University of Cincinnati College of Medicine, Cincinnati, Ohio, USA; Vanderbilt University Medical Center, Nashville, Tennessee, USA; Coronavirus and Other Respiratory Viruses Division, National Center for Immunization and Respiratory Diseases, Centers for Disease Control and Prevention, Atlanta, Georgia, USA; Coronavirus and Other Respiratory Viruses Division, National Center for Immunization and Respiratory Diseases, Centers for Disease Control and Prevention, Atlanta, Georgia, USA; Coronavirus and Other Respiratory Viruses Division, National Center for Immunization and Respiratory Diseases, Centers for Disease Control and Prevention, Atlanta, Georgia, USA; Vanderbilt University Medical Center, Nashville, Tennessee, USA; Influenza Division, National Center for Immunization and Respiratory Diseases, Centers for Disease Control and Prevention, Atlanta, Georgia, USA; Vanderbilt University Medical Center, Nashville, Tennessee, USA

**Keywords:** influenza, oseltamivir, pediatrics, antivirals, guideline-concordant

## Abstract

**Background:**

Guidelines state that all hospitalized children with suspected or confirmed influenza receive prompt treatment with influenza-specific antivirals. We sought to determine the frequency of, and factors associated with, antiviral receipt among hospitalized children.

**Methods:**

We conducted active surveillance of children presenting with fever or respiratory symptoms from 1 December 2016 to 31 March 2020 at 7 pediatric medical centers in the New Vaccine Surveillance Network. The cohort consisted of children hospitalized with influenza A or B confirmed by clinical or research testing. The primary outcome was frequency of antiviral receipt during hospitalization. We used logistic regression to obtain adjusted odds ratios (aORs) and 95% confidence intervals (CIs) for factors associated with antiviral receipt.

**Results:**

A total of 1213 children with laboratory-confirmed influenza were included. Overall, 652 children (53.8%) received an antiviral. Roughly 63.0% of children received clinical influenza testing. Among those with clinical testing, 67.4% received an antiviral. Factors associated with higher odds of antiviral receipt included hematologic (aOR = 1.76; 95% CI = 1.03–3.02) or oncologic/immunocompromising (aOR = 2.41; 95% CI = 1.13–5.11) disorders, prehospitalization antiviral receipt (aOR = 2.34; 95% CI = 1.49–3.67), clinical influenza testing (aOR = 3.07; 95% CI = 2.28–4.14), and intensive care unit admission (aOR = 1.53; 95% CI = 1.02–2.29). Symptom duration >2 days was associated with lower odds of antiviral treatment (aOR = 0.40; 95% CI = .30–.52). Antiviral receipt varied by site with a 5-fold difference across sites.

**Conclusions:**

Almost half of children hospitalized with influenza did not receive antivirals. Additional efforts to understand barriers to guideline adherence are crucial for optimizing care in children hospitalized with influenza.

## Teaser

Almost half of children hospitalized with influenza did not receive antiviral treatment. Antiviral treatment varied according to several individual- and system-level factors. Further study is needed to improve appropriate and timely antiviral treatment in hospitalized children.

Influenza illness accounts for up to 10% of all pediatric hospitalizations during the winter season in the United States [1, 2]. While young children and those with comorbidities are at increased risk for influenza complications, the majority of children hospitalized for influenza are otherwise healthy [3]. Complications of influenza include conditions that require hospitalization, such as pneumonia, myocarditis, myositis, and encephalitis; influenza can also lead to multiorgan failure and even death [2, 4–8].

Meta-analyses of clinical trials demonstrate that influenza antiviral use results in shorter symptom duration and lower risk of developing subsequent pneumonia, sinusitis, and otitis media infections [2, 9–11]. Observational studies also support outpatient use of influenza-specific antivirals, with antiviral use associated with decreased hospitalizations and influenza-related complications [12–15]. Among hospitalized children, early use of antivirals may decrease the length of hospital stay, use of mechanical ventilation, and intensive care unit (ICU) admission [16–18].

The American Academy of Pediatrics (AAP), Infectious Diseases Society of America (IDSA), and Centers for Disease Control and Prevention (CDC) recommend initiation of influenza antiviral treatment as soon as possible in all children hospitalized with confirmed or suspected influenza, regardless of symptom duration, vaccination status, or underlying risk factors [19–21]. However, recent evidence suggests that the use of antivirals in the pediatric inpatient setting is highly variable and suboptimal, with a substantial number of children not receiving guideline-concordant antiviral treatment [4, 22–24]. Prior studies that sought to determine factors that contribute to variable antiviral use lacked laboratory confirmation of influenza and did not account for important factors such as outpatient antiviral use, vaccination status, and symptom duration [22, 24].

A better understanding of the drivers of influenza antiviral use will inform future efforts to improve the clinical management of children hospitalized with influenza illness. Therefore, the objectives of this study were to determine the prevalence of and factors associated with influenza antiviral receipt among children hospitalized with laboratory-confirmed influenza illness in a multicenter, prospective surveillance cohort in the United States.

## METHODS

### Study Design and Population

Data from the CDC-funded New Vaccine Surveillance Network (NVSN) were used for this study. The NVSN is a prospective, active, population-based surveillance platform of children with acute respiratory illness across 7 pediatric medical centers in the following US cities: Cincinnati, Ohio; Houston, Texas; Kansas City, Missouri; Nashville, Tennessee; Pittsburgh, Pennsylvania; Rochester, New York; and Seattle, Washington [25, 26]. Children were eligible for enrollment if they were aged <18 years and had an illness duration of <14 days, had at least 1 qualifying acute respiratory illness sign or symptom (eg, apnea, cough, earache, fever, myalgia, nasal congestion, runny nose, sore throat, vomiting after coughing, shortness of breath [rapid or shallow breathing]), wheezing, or apparent life-threatening event or brief resolved unexplained event), and resided in a surveillance site area. Data for this study were restricted to the dates of 1 December 2016 to 31 March 2020. Children with fever and neutropenia associated with malignancy, those readmitted within 4 days, or those transferred from another hospital after an admission of >48 hours were excluded from NVSN enrollment. Written informed consent was obtained from the parent or guardian, and assent was obtained when applicable. The institutional review boards at the CDC and each of the 7 surveillance sites approved this study. This report was prepared in compliance with the STrengthening the Reporting of OBservational studies in Epidemiology (STROBE) guidelines [27].

### Definition of Influenza Virus Infection

Influenza cases were defined as those with laboratory-confirmed influenza, which included either a positive clinical or research test for influenza virus A or B. Clinical testing included antigen-based and molecular assays reported in the electronic medical record at each of the 7 sites. In addition to clinical testing, study personnel collected mid-turbinate nasal or oropharyngeal swabs from all enrolled children for research testing (results not reported to providers). For intubated patients, tracheal aspirates were accepted as an alternative specimen source. When nasal, oropharyngeal, or tracheal aspirate specimens were not collected, clinically salvaged respiratory specimens were obtained. These research specimens were tested at each site using commercial or institution-specific in-house reverse-transcription polymerase chain reaction (RT-PCR) assays for influenza. Diagnostic assay methods varied by site and included the Luminex NxTAG Respiratory Pathogen Panel (Cincinnati and Kansas City), BioFire FilmArray Respiratory Panel (Seattle), Applied Biosystems TaqMan Array Microfluidic Card (Rochester), and in-house RT-PCR assays (Houston, Pittsburgh, and Nashville) [28]. All sites conducted CDC-sponsored proficiency testing to ensure valid and consistent respiratory viral detection [28, 29].

### Children at Increased Risk for Influenza Complications

Children at increased risk for influenza complications were identified using the 2018 IDSA Clinical Practice Guidelines definition for children at increased risk of influenza and included age <5 years (especially <2 years), underlying chronic pulmonary (including asthma), cardiovascular, renal, hepatic, gastrointestinal, hematologic (including sickle cell disease), oncologicor immunosuppressive, metabolic (including diabetes mellitus), and neurologic and neuromuscular conditions [30]. Underlying conditions were collected from electronic medical records at each site. Other conditions at high risk for influenza complications, including children with long-term aspirin use, those living in a chronic care facility, and pregnant children, were not included as part of the increased risk definition as these conditions were either uncommon in our dataset or the necessary data were unavailable for assessment.

### Outcomes

The primary outcome was proportion of antiviral use, defined as the number of children with with influenza confirmed by research or routine clinical laboratory testing who received an influenza antiviral (oseltamivir, peramivir, baloxavir, or zanamivir) during hospitalization divided by the total number of children hospitalized with influenza. Our secondary outcomes were factors associated with in-hospital influenza antiviral receipt.

### Statistical Analyses

Descriptive statistics are presented as medians (interquartile ranges [IQRs]) for continuous variables and frequencies (percentages) for categorical variables. The proportion of children with antiviral receipt was calculated by dividing the number of hospitalized children who received an antiviral by the total number of children hospitalized with influenza confirmed by research or routine clinical laboratory testing. To account for missing data in the regression model, we used multiple imputation by chained equations with M = 20 iterations as implemented in the “mice” package in R. Adjusted odds ratios (aORs) and corresponding 95% confidence intervals (CIs) for factors potentially associated with antiviral receipt were generated using mixed-effects logistic regression that included the following fixed-effects covariates: age; sex; comorbidities; symptom duration at presentation, defined as time between self-reported symptom onset and admission; presence of gastrointestinal symptoms; seasonal influenza vaccination status; receipt of antivirals in the outpatient setting prior to hospitalization; clinical testing for influenza; clinical influenza co-detection; early admission to the ICU; influenza season; and admission during peak influenza season. Individuals were captured at the encounter level, allowing for multiple encounters for each individual across influenza seasons. However, outcomes of encounters that come from the same individual may be correlated due to patient-specific factors not accounted for by the model. To account for this, we included patient-level identifiers (to account for repeat encounters across influenza seasons) and study site as random effects in the model. Influenza season was defined as the period spanning the first Sunday in July for a given calendar year until the day before the first Sunday in July of the subsequent year. Peak influenza season was defined as the 13 consecutive weeks (with Sunday as the index day) with the highest cumulative number of influenza cases at each site [7, 31]. All analyses were performed using R, version 4.4.1.

To compare heterogeneity in antiviral receipt by those at increased risk for influenza complications (defined based on 2018 IDSA Clinical Practice Guidelines as age <5 years or presence of an underlying condition) [30], we used our mixed-effects logistic regression model to obtain patient-specific predicted values (or expected receipt) for the probability of antiviral use to compare with actual receipt. We used descriptive statistics to compare the distribution of predicted probabilities between those at increased risk for influenza complications and those not at increased risk for influenza complications.

## RESULTS

### Study Population

**Figure 1.**
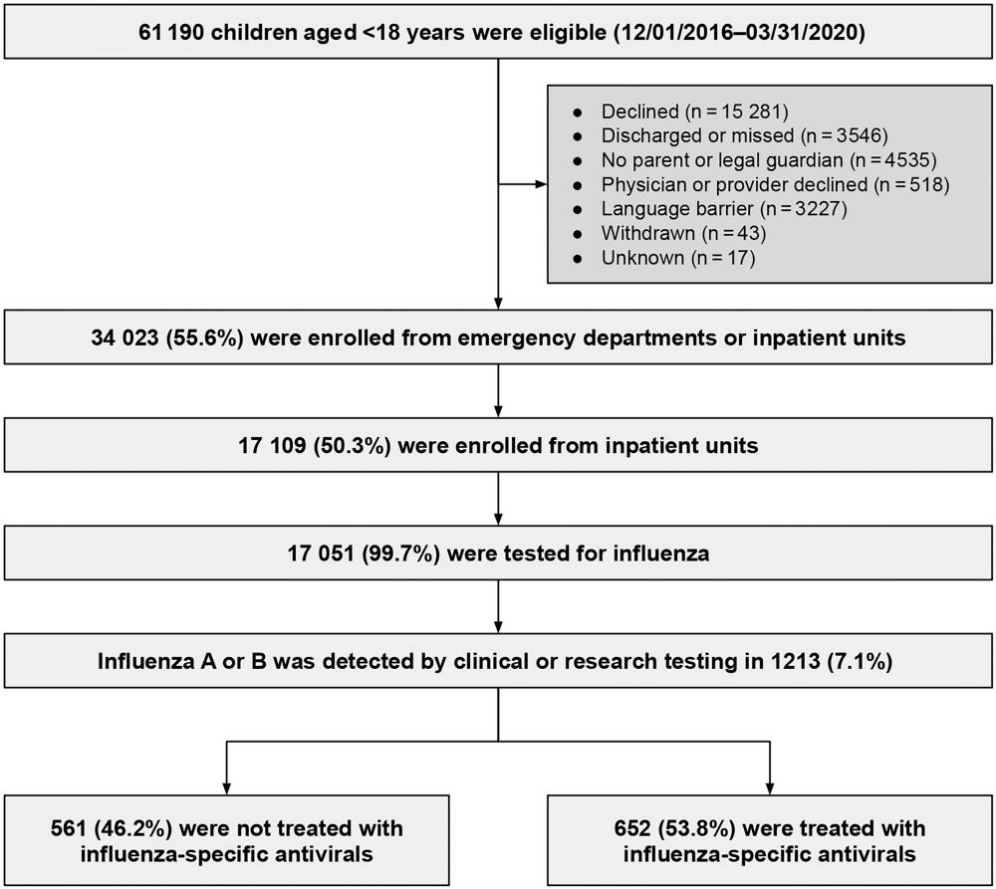
Flowchart of study participants.

Of 17 109 hospitalized children enrolled over the study period, 17 051 (99.7%) underwent influenza testing, 1213 (7.1%, representing 1195 children with 1 encounter and 9 with 2 encounters) of whom were positive for influenza A or B (Figure 1). A total of 162 (13.4%) children were identified by clinical testing only, 602 (49.6%) by clinical and research testing, and 449 (37.0%) by research testing only. The median age of confirmed cases was 3.7 years (IQR, 1.3–8.1). Most were male (55.6%), had public insurance (64.3%), and did not receive antivirals prior to admission (89.9%); 41.2% were non-Hispanic white, 27.7% were non-Hispanic Black, 20.4% were Hispanic, and 10.7% were another race (Table 1).

**Table 1.** Demographic and Clinical Characteristics of Hospitalized Children Who Tested Positive for Influenza A or B by Clinical or Research Assays Stratified by Influenza-Specific Antiviral Receipt, New Vaccine Surveillance Network, 1 December 2016 to 31 March 2020

Characteristic	All (n = 1213)	No Antiviral Receipt (n = 561)	Antiviral Receipt (n = 652)
Age at presentation, median (IQR), y	3.7 (1.3–8.1)	3.7 (1.3–7.8)	3.6 (1.2–8.4)
Age group at presentation, n (%), y			
0–1	415 (34.2)	179 (31.9)	236 (36.2)
2–4	289 (23.8)	152 (27.1)	137 (21.0)
5–17	509 (42.0)	230 (41.0)	279 (42.8)
Sex, n (%)			
Female	538 (44.4)	240 (42.8)	298 (45.7)
Male	675 (55.6)	321 (57.2)	354 (54.3)
Race and Hispanic origin, n (%)			
Hispanic	246/1205 (20.4)	81/560 (14.5)	165/645 (25.6)
Non-Hispanic Black	334/1205 (27.7)	154/560 (27.5)	180/645 (27.9)
Non-Hispanic White	496/1205 (41.2)	265/560 (47.3)	231/645 (35.8)
Non-Hispanic other	129/1205 (10.7)	60/560 (10.7)	69/645 (10.7)
Insurance status, n (%)			
Private	348/1191 (29.2)	172/551 (31.2)	176/640 (27.5)
Public	766/1191 (64.3)	351/551 (63.7)	415/640 (64.8)
Both	20/1191 (1.7)	9/551 (1.6)	11/640 (1.7)
Self-pay	57/1191 (4.8)	19/551 (3.4)	38/640 (5.9)
Risk factor for influenza complications, n (%)			
Any risk factor	1073 (88.5)	473 (84.3)	600 (92.0)
Aged 0–4 y	704 (58.0)	331 (59.0)	373 (57.2)
Respiratory disorder	362 (29.8)	151 (26.9)	211 (32.4)
Cardiovascular disorder	99 (8.2)	34 (6.1)	65 (10.0)
Neurologic or neuromuscular disorder	187 (15.4)	77 (13.7)	110 (16.9)
Hematologic disorder	91 (7.5)	23 (4.1)	68 (10.4)
Oncologic or immunocompromising disorder	56 (4.6)	11 (2.0)	45 (6.9)
Endocrine disorder	62 (5.1)	25 (4.5)	37 (5.7)
Renal or urologic disorder	26 (2.1)	11 (2.0)	15 (2.3)
Gastrointestinal or hepatic disorder	148 (12.2)	50 (8.9)	98 (15.0)
Genetic or metabolic disorder	202 (16.7)	80 (14.3)	122 (18.7)
Signs and symptoms			
Days symptomatic at presentation, median (IQR)	3.0 (2.0–5.0)	4.0 (3.0–6.0)	3.0 (2.0–4.0)
1 or 2, n (%)	439/1207 (36.4)	137/559 (24.5)	302/648 (46.6)
3 or more, n (%)	768/1207 (63.6)	422/559 (75.5)	346/648 (53.4)
Fever, n (%)	1129/1206 (93.6)	525/555 (94.6)	604/651 (92.8)
Cough, n (%)	1139 (93.9)	531 (94.7)	608 (93.3)
Congestion or runny nose, n (%)	1061/1210 (87.7)	487/559 (87.1)	574/651 (88.2)
Sore throat, n (%)	468/905 (51.7)	216/429 (50.3)	252/476 (52.9)
Dyspnea, n (%)	811/1201 (67.5)	365/553 (66.0)	446/648 (68.8)
Myalgia, n (%)	354/825 (42.9)	165/392 (42.1)	189/433 (43.6)
Chills, n (%)	567/1101 (51.5)	255/506 (50.4)	312/595 (52.4)
Vomiting, n (%)	419/1207 (34.7)	188/556 (33.8)	231/651 (35.5)
Diarrhea, n (%)	371/1205 (30.8)	194/557 (34.8)	177/648 (27.3)
Antiviral receipt, n (%)			
Prior receipt of influenza-specific antivirals	122/1204 (10.1)	46/554 (8.3)	76/650 (11.7)
Receipt of in-hospital antivirals on day 1 or 2	598/649 (92.1)	0/0 (NA)	598/649 (92.1)
Received current season influenza vaccine, n (%)	565 (46.6)	249 (44.4)	316 (48.5)
Clinical testing,^a^n (%)			
Not tested for influenza	396 (32.6)	263 (46.9)	133 (20.4)
Tested negative for influenza	53 (4.4)	46 (8.2)	7 (1.1)
Tested positive for influenza	764 (63.0)	252 (44.9)	512 (78.5)
Research testing, n (%)			
Not tested for influenza	22 (1.8)	5 (0.9)	17 (2.6)
Tested negative for influenza	140 (11.5)	52 (9.3)	88 (13.5)
Tested positive for influenza	1051 (86.6)	504 (89.8)	547 (83.9)
Clinical influenza co-detection, n (%)	85/760 (11.2)	31/250 (12.4)	54/510 (10.6)
Length of stay, median (IQR), d	2.0 (1.0–3.0)	1.0 (1.0–2.0)	2.0 (1.0–3.0)
Intensive care unit admission, n (%)			
Day 1 or 2	163 (13.4)	51 (9.1)	112 (17.2)
Day 3 or after	13 (1.1)	4 (0.7)	9 (1.4)
Influenza season, n (%)			
2016–2017	215 (17.7)	111 (19.8)	104 (16.0)
2017–2018	296 (24.4)	128 (22.8)	168 (25.8)
2018–2019	285 (23.5)	130 (23.2)	155 (23.8)
2019–2020	417 (34.4)	192 (34.2)	225 (34.5)
Peak influenza season, n (%)	994 (81.9)	453 (80.7)	541 (83.0)
Study site, n (%)			
A	103 (8.5)	76 (13.5)	27 (4.1)
B	193 (15.9)	67 (11.9)	126 (19.3)
C	135 (11.1)	73 (13.0)	62 (9.5)
D	105 (8.7)	32 (5.7)	73 (11.2)
E	209 (17.2)	55 (9.8)	154 (23.6)
F	100 (8.2)	49 (8.7)	51 (7.8)
G	368 (30.3)	209 (37.3)	159 (24.4)

Abbreviation: IQR, interquartile range.

^a^Clinical tests performed >1 day after antiviral receipt were excluded.

### Prevalence and Timing of Antiviral Receipt

Of 1213 hospitalized children with laboratory-confirmed influenza, 652 (53.8%) received an antiviral. Among the 561 (46.2%) children who did not receive an antiviral, 263 (46.9%) were not clinically tested for influenza. Among those who underwent a clinical influenza test, 519 of 817 (63.5%) received an antiviral, and among those who tested positive clinically, 512 of 764 (67.0%) received an antiviral. Among 153 infants aged <6 months (ineligible for vaccination), 96 (62.7%) received an antiviral. Compared with children not clinically tested, those clinically tested more frequently received antivirals (63.5% vs 33.6%), time between self-reported symptom onset and admission was ≤2 days (40.2% vs 28.4%), and a lower proportion of children clinically tested were non-Hispanic white (33.5% vs 57.0%; Supplementary Table 1).

**Figure 2.**
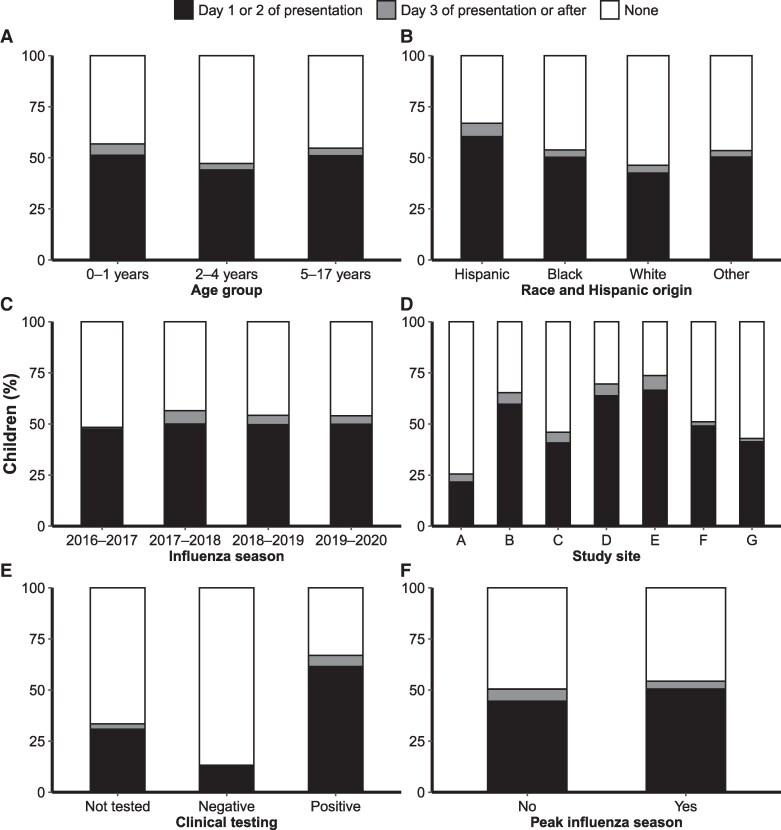
Frequency and timing of antiviral receipt in hospitalized children with influenza enrolled in the New Vaccine Surveillance Network (1 December 2016 to 31 March 2020), stratified by age group (*A*), race and Hispanic origin (*B*), influenza season (*C*), study site (*D*), clinical testing (*E*), and peak influenza season (*F*). Day refers to the day of hospitalization on which the first dose of antiviral was received. None refers to cases where no antiviral was received during the hospitalization.

Among those who received an antiviral, 1 child received peramivir only, 650 received oseltamivir only, and 1 received both peramivir and oseltamivir. The date of antiviral receipt was recorded for 649 children (99.5%): 598 (92.1%) received an antiviral on day 1 or 2 of presentation and 51 (7.9%) received an antiviral on or after day 3. Antiviral receipt was highest among Hispanic children (67.1%), and the site-specific frequency of antiviral receipt ranged from 26.2% to 73.7% (Figure 2). The proportion of antiviral receipt was generally higher with shorter time between self-reported symptom onset and admission (Supplementary Figure 1).

### Factors Associated With Antiviral Receipt Among Children With Influenza

**Figure 3.**
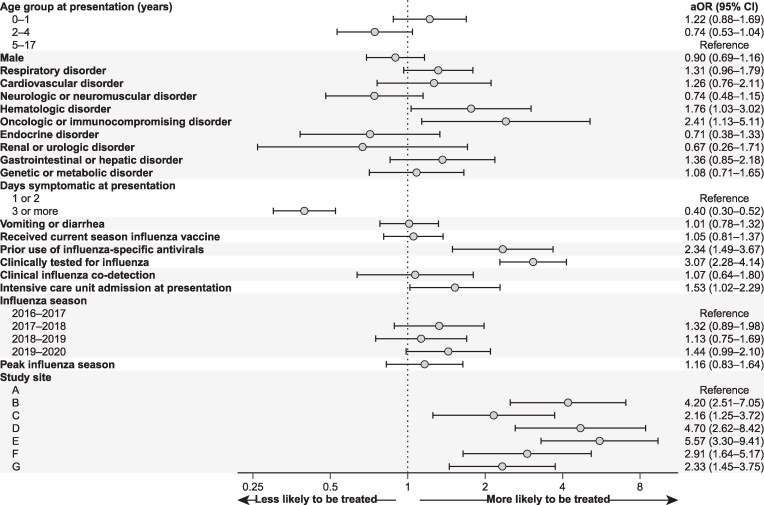
Factors associated with antiviral receipt among hospitalized children with influenza enrolled in the New Vaccine Surveillance Network (1 December 2016 to 31 March 2020) identified from a generalized linear mixed-effects model with a patient-level identifier and study site as random effects. Abbreviations: aOR, adjusted odds ratio; CI, confidence interval.

Patient-level factors associated with higher odds of antiviral receipt included a hematologic disorder (aOR = 1.76; 95% CI = 1.03–3.02), an oncologic or immunocompromising disorder (aOR = 2.41; 95% CI = 1.13–5.11), receipt of antivirals for current illness prior to hospitalization (aOR = 2.34; 95% CI = 1.49–3.67), clinical testing for influenza (aOR = 3.07; 95% CI = 2.28–4.14), and ICU admission at presentation (aOR = 1.53; 95% CI = 1.02–2.29; Figure 3). Time between self-reported symptom onset and admission >2 days at presentation was associated with lower odds of antiviral receipt (aOR = 0.40; 95% CI .30–.52). System-level factors, such as study site, were also associated with receipt of an antiviral (Figure 3). We identified significant heterogeneity in odds of antiviral receipt by site, with the odds of receipt differing by an estimated 457% difference between the sites with the highest (Site E) and lowest (Site A) receipt (aOR = 5.57; 95% CI 3.30–9.41).

**Figure 4.**
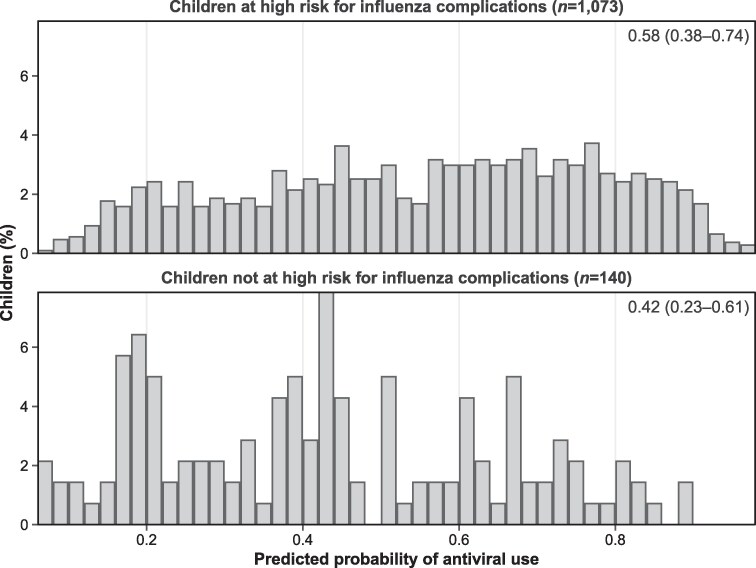
Distribution of the predicted probability of antiviral receipt among children disaggregated by the risk of influenza complications (according to the 2018 Infectious Diseases Society of America Clinical Practice Guideline). The group-specific median and interquartile range are indicated in the top right corner of each subfigure.

Figure 4 shows the distribution of the predicted probability of antiviral receipt based on the regression model, stratified by the risk for influenza complications (based on 2018 IDSA guidelines). While there was a higher tendency for those at increased risk for influenza complications to receive an antiviral compared with those not at increased risk, our model identifies a large proportion of children at increased risk as being unlikely to receive an antiviral. As shown in Figure 4, the median expected antiviral receipt among children deemed at increased risk was 58%. Among those not at increased risk for influenza complications, the median expected receipt was 42%. Additionally, Figure 4 illustrates that our model covariates selected a priori explain a large degree of variation in actual antiviral receipt within both groups defined by increased risk status.

We performed a secondary analysis of factors associated with antiviral receipt among 764 children with laboratory-confirmed influenza by rapid or RT-PCR clinical testing. Results were consistent with the primary analysis with the exception of prior antiviral testing, which was no longer a significant factor (Supplementary Figure 2).

## DISCUSSION

In this large, multicenter, prospective study of antiviral use among hospitalized children with laboratory-confirmed influenza, we found that almost half did not receive antiviral therapy despite widely endorsed recommendations by national organizations and treatment guidelines. Importantly, almost 37% of hospitalized infants in our study aged <6 months who are ineligible for influenza vaccination and at highest risk for severe influenza and influenza-related complications [8, 31–33] did not receive an antiviral. Prescribing was also higher among those clinically tested for influenza, those who received antivirals prior to hospitalization, those with an underlying hematologic disorder or an oncologic/immunocompromising disorder, and those admitted to the ICU at presentation. Conversely, antiviral use was less frequent when symptoms had been present for >2 days at hospitalization and at some study sites. Our findings underscore the need for increased efforts to improve antiviral use in managing influenza disease among hospitalized children.

Our finding of limited use of antivirals in hospitalized children with influenza illness is concerning. The IDSA, CDC, and AAP recommend treatment as soon as possible for hospitalized children with suspected or confirmed influenza [20, 34, 35]. These guidelines also state that lack of influenza testing or delays in obtaining testing results should not delay empiric antiviral treatment in this population. Our results align with those from our previous study in which we used NVSN data from the 2015–2016 influenza season and found that 52% of hospitalized children with a positive clinical test for influenza received antiviral treatment [36]. Our current analysis indicates that there was minimal change in the subsequent years; adherence to guidelines for antiviral use in hospitalized children remains suboptimal. The reasons for lack of treatment of children hospitalized with symptomatic influenza illness are unclear. Oseltamivir allergy and serious adverse events (ie, neuropsychiatric events [7, 23] or Stevens–Johnson syndrome [35, 37]) are generally thought to be rare. However, if a child does have an oseltamivir allergy, an alternative antiviral such as peramivir (or potentially baloxavir) may be used. Given changes in healthcare utilization for influenza and influenza transmission following the coronavirus disease 2019 pandemic, continued monitoring of influenza antiviral use is warranted [38–40]. To date, the CDC recommends empiric treatment with influenza antivirals during influenza season for those who are at high risk of severe influenza, and treatment should not be delayed for clinical testing results despite other circulating virus strains (such as severe acute respiratory syndrome coronavirus 2) [41]. However, this guidance may change in the future based on epidemiologic trends of these viruses.

There was varied receipt of antivirals across the 7 geographically distinct study sites, ranging from 26.2% to 73.7%. Among those with clinical influenza testing, 67% received an antiviral. This is similar to findings from 36 academic children's hospitals between 2007 and 2020 that showed median oseltamivir use of 66% among those hospitalized with an influenza diagnosis [22]. In the most recent season of that study, oseltamivir use by hospital ranged from 56.5% to 90.1%. The reasons for this high variation are likely multifactorial. Differences in institutional cultures, presence of standardized care protocols, and location of medical training have all been shown to play a role in variation in care for other disease processes. It is also likely that, despite strong national recommendations for treatment, a misperception regarding evidence for antiviral treatment in hospitalized children is a factor in the variation of influenza treatment.

A recent meta-analysis of randomized trials in hospitalized adults suggests that antivirals shorten hospital length of stay (LOS) [42]. However, data for hospitalized children are primarily driven by observational studies. A study of a prospectively derived cohort of children hospitalized with laboratory-confirmed influenza found that early oseltamivir receipt was associated with a shorter LOS among children at increased risk for influenza complications and, separately, in those admitted to the ICU [17]. However, children who received oseltamivir ≥3 days after symptom onset did not have a significantly lower LOS compared with those who did not receive antiviral therapy. These data are supported by retrospective studies that have also shown that antiviral use is associated with a shortened hospital course in adults [43], as well as hospitalized children at increased risk of influenza complications [16] and those with severe disease admitted to the ICU [44–46]. A recent retrospective cohort study found that early oseltamivir treatment was associated with shorter LOS, transfer to the ICU, a composite outcome of death/extracorporeal membrane oxygenation use, and readmission among all children admitted with influenza. These findings were similar among those with and without underlying comorbidities [18]. However, that study could not account for confounding factors such as symptom duration, vaccination status, or antiviral receipt prior to hospitalization.

While observational studies suggest limited benefit in LOS with antivirals among those with symptoms that last <3 days, there are no randomized trials to confirm these findings. However, there is evidence of a clinical benefit of antivirals even when initiated after 48 hours of symptoms [8, 41–44, 47–52], and there are hospital outcomes other than LOS where antivirals may be beneficial to hospitalized patients, such as symptom severity, respiratory support, and development of influenza complications. Additional studies are needed on these outcomes to inform clinical decision-making. Most children hospitalized for influenza are otherwise healthy, and there are no randomized trials of antiviral treatment in this population. Rigorous studies are needed in healthy children hospitalized for influenza to determine the effectiveness of guideline treatment decisions, especially surrounding the timing of treatment in relation to disease onset.

We identified several factors associated with low antiviral use that may serve as targets for interventions to improve the care of children hospitalized with influenza. First, children with clinical influenza testing had 3-fold higher odds of antiviral receipt than those without clinical testing. In our cohort, approximately 47% of untreated children did not receive clinical influenza testing. However, almost 64% of those with a clinical influenza test and 67% of those with a positive clinical test for influenza received an antiviral. The IDSA guidelines recommend influenza testing in all hospitalized individuals with acute respiratory illness [30]. While establishing a diagnosis through testing is not a requirement for, and should not delay, antiviral treatment in hospitalized children, it is likely that efforts to improve appropriate clinical testing or to increase provider suspicion for influenza infection will result in more antiviral receipt.

Second, outpatient antiviral receipt prior to hospitalization was a strong predictor of inpatient antiviral receipt. Recent evidence suggests that the receipt of outpatient influenza-specific antivirals, including among children at increased risk for influenza complications and those aged <2 years, is low [53, 54]. Interventions to improve early antiviral receipt in the outpatient setting will likely aid in overcoming clinical inertia to treat in the inpatient setting.

Third, children who presented on or after day 3 of symptoms were significantly less likely to receive antivirals compared with those who presented on day 1 or 2. Qualitative research by the CDC on antiviral prescribing practices revealed that there is a wide range of physician perceptions as to the effectiveness of influenza-specific antivirals and that some clinicians may not prescribe antivirals after the 2-day window, which was found to be optimal treatment timing in clinical trials [55]. The low proportion of antiviral receipt in children with >2 days of symptoms may reflect either a misunderstanding of national recommendations or a belief that antiviral treatment is not effective after 2 days of symptoms in those with severe disease. Future interventions could focus on increasing antiviral use in hospitalized children with >2 days of symptoms regardless of duration of symptoms.

Our results must be considered in the context of several limitations. The study was performed at 7 academic children's hospitals, and findings may not be generalizable to community hospital settings. Clinical testing information was determined by chart review at the treating institution, and we may have underestimated clinical testing if it was performed at an outside facility and written documentation of the testing was not provided to the treating hospital. Additionally, receipt of an antiviral was well captured in our study, but we could not determine if an individual received the full antiviral treatment course or the time of day of individual doses. It is possible that some children were suboptimally treated with antivirals. Finally, time stamps (or clock times) were not available in our study to measure time in hours. Our approach of measuring symptom duration at presentation in days rather than hours may lead to an overestimation of the interval between symptom onset and presentation (as partial days are counted in full). Therefore, we may have overestimated the proportion of antiviral receipt in those with <48 hours of symptoms. Despite these limitations, our findings provide strong evidence that the management of children hospitalized for influenza can be improved through improving guideline-concordant antiviral treatment.

## CONCLUSIONS

Among hospitalized children with laboratory-confirmed influenza, approximately half received guideline-recommended influenza-specific antivirals. Antiviral receipt varied according to several individual-and system-level factors, including specific underlying conditions, clinical testing, prior antiviral treatment, symptom duration, early ICU admission, and treatment site. Future studies could focus on identifying barriers to treatment and improving clinical influenza testing, providing antiviral treatment for children with influenza-associated hospitalizations with >2 days of symptoms, and standardizing care both within and across institutions.

## Supplementary Material

ciae573_Supplementary_Data

## Supplementary Data

Supplementary materials are available at *Clinical Infectious Diseases* online. Consisting of data provided by the authors to benefit the reader, the posted materials are not copyedited and are the sole responsibility of the authors, so questions or comments should be addressed to the corresponding author.

## Note

***Financial support.*** This study was supported by cooperative agreement IP16-004 with the Centers for Disease Control and Prevention. J. W. A. (K23 AI168496) was partially supported by the National Institute for Allergy and Infectious Diseases of the National Institutes of Health.

## References

[1] Rolfes MA, Foppa IM, Garg S, et al Annual estimates of the burden of seasonal influenza in the United States: a tool for strengthening influenza surveillance and preparedness. Influenza Other Respir Viruses 2018; 12:132–7. 29446233 10.1111/irv.12486 PMC5818346

[2] Wolf RM, Antoon JW. Influenza in children and adolescents: epidemiology, management, and prevention. Pediatr Rev 2023; 44:605–17. 37907421 10.1542/pir.2023-005962 PMC10676733

[3] Centers for Disease Control and Prevention . FluView interactive. Available at:https://www.cdc.gov/fluview/index.html. Accessed 19 December 2023.

[4] Antoon JW, Hall M, Herndon A, et al Prevalence, risk factors, and outcomes of influenza-associated neurologic complications in children. J Pediatr 2021; 239:32–8.e5. 34216629 10.1016/j.jpeds.2021.06.075 PMC8604779

[5] Grijalva CG, Zhu Y, Williams DJ, et al Association between hospitalization with community-acquired laboratory-confirmed influenza pneumonia and prior receipt of influenza vaccination. JAMA 2015; 314:1488–97. 26436611 10.1001/jama.2015.12160 PMC4688454

[6] Gill PJ, Ashdown HF, Wang K, et al Identification of children at risk of influenza-related complications in primary and ambulatory care: a systematic review and meta-analysis. Lancet Respir Med 2015; 3:139–49. 25481379 10.1016/S2213-2600(14)70252-8

[7] Antoon JW, Williams DJ, Bruce J, et al Population-based incidence of influenza-associated serious neuropsychiatric events in children and adolescents. JAMA Pediatr 2023; 177:967–9. 37486679 10.1001/jamapediatrics.2023.2304 PMC10366945

[8] Quertermous BP, Williams DJ, Bruce J, et al Incidence of influenza-associated neurologic and psychiatric complications requiring hospitalization in children ages 5–17 years. Pediatr Infect Dis J 2024; 43:959–62. 38869312 10.1097/INF.0000000000004424 PMC11408088

[9] Malosh RE, Martin ET, Heikkinen T, Brooks WA, Whitley RJ, Monto AS. Efficacy and safety of oseltamivir in children: systematic review and individual patient data meta-analysis of randomized controlled trials. Clin Infect Dis 2018; 66:1492–500. 29186364 10.1093/cid/cix1040

[10] Dobson J, Whitley RJ, Pocock S, Monto AS. Oseltamivir treatment for influenza in adults: a meta-analysis of randomised controlled trials. Lancet 2015; 385:1729–37. 25640810 10.1016/S0140-6736(14)62449-1

[11] Liu JW, Lin SH, Wang LC, Chiu HY, Lee JA. Comparison of antiviral agents for seasonal influenza outcomes in healthy adults and children: a systematic review and network meta-analysis. JAMA Netw Open 2021; 4:e2119151. 34387680 10.1001/jamanetworkopen.2021.19151 PMC8363918

[12] Lee JJ, Smith M, Bankhead C, et al Oseltamivir and influenza-related complications in children: a retrospective cohort in primary care. Eur Respir J 2020; 56:1902246. 32527739 10.1183/13993003.02246-2019

[13] Piedra PA, Schulman KL, Blumentals WA. Effects of oseltamivir on influenza-related complications in children with chronic medical conditions. Pediatrics 2009; 124:170–8. 19564297 10.1542/peds.2008-0977

[14] Falagas ME, Koletsi PK, Vouloumanou EK, Rafailidis PI, Kapaskelis AM, Rello J. Effectiveness and safety of neuraminidase inhibitors in reducing influenza complications: a meta-analysis of randomized controlled trials. J Antimicrob Chemother 2010; 65:1330–46. 20488984 10.1093/jac/dkq158

[15] Dai Z, Zhang L, Yu Q, Liu L, Yang M, Fan K. Early administration of oseltamivir within 48 hours after onset of flulike symptoms can reduce the risk of influenza B virus-associated pneumonia in hospitalized pediatric patients with influenza B virus infection. Pediatr Infect Dis J 2020; 39:e20–2. 31929434 10.1097/INF.0000000000002528

[16] Miyakawa R, Barreto NB, Kato RM, Neely MN, Russell CJ. Early use of anti-influenza medications in hospitalized children with tracheostomy. Pediatrics 2019; 143:e20182608. 30814271 10.1542/peds.2018-2608 PMC6398370

[17] Campbell AP, Tokars JI, Reynolds S, et al Influenza antiviral treatment and length of stay. Pediatrics 2021; 148:e2021050417. 34470815 10.1542/peds.2021-050417

[18] Walsh PS, Schnadower D, Zhang Y, Ramgopal S, Shah SS, Wilson PM. Association of early oseltamivir with improved outcomes in hospitalized children with influenza, 2007–2020. JAMA Pediatr 2022; 176:e223261. 36121673 10.1001/jamapediatrics.2022.3261 PMC9486642

[19] Committee on Infectious Diseases . Recommendations for prevention and control of influenza in children, 2022–2023. Pediatrics 2022; 150:e2022059274. 36065749 10.1542/peds.2022-059274

[20] Uyeki TM, Bernstein HH, Bradley JS, et al Clinical practice guidelines by the Infectious Diseases Society of America: 2018 update on diagnosis, treatment, chemoprophylaxis, and institutional outbreak management of seasonal influenza. Clin Infect Dis 2019; 68:895–902. 30834445 10.1093/cid/ciy874 PMC6769232

[21] Centers for Disease Control and Prevention . Influenza antiviral medications: summary for clinicians. Available at: https://www.cdc.gov/flu/hcp/antivirals/summary-clinicians.html. Accessed 19 December 2023.

[22] Walsh PS, Schnadower D, Zhang Y, Ramgopal S, Shah SS, Wilson PM. Assessment of temporal patterns and patient factors associated with oseltamivir administration in children hospitalized with influenza, 2007–2020. JAMA Netw Open 2022; 5:e2233027. 36149655 10.1001/jamanetworkopen.2022.33027 PMC9508650

[23] Harrington R, Adimadhyam S, Lee TA, Schumock GT, Antoon JW. The relationship between oseltamivir and suicide in pediatric patients. Ann Fam Med 2018; 16:145–8. 29531106 10.1370/afm.2183 PMC5847353

[24] Stockmann C, Byington CL, Pavia AT, et al Limited and variable use of antivirals for children hospitalized with influenza. JAMA Pediatr 2017; 171:299–301. 28114638 10.1001/jamapediatrics.2016.3484

[25] Perez A, Lively JY, Curns A, et al Respiratory virus surveillance among children with acute respiratory illnesses—new vaccine surveillance network, United States, 2016–2021. MMWR Morb Mortal Wkly Rep 2022; 71:1253–9. 36201373 10.15585/mmwr.mm7140a1 PMC9541034

[26] Antoon JW, Stopczynski T, Amarin JZ, et al Accuracy of influenza ICD-10 diagnosis codes in identifying influenza illness in children. JAMA Netw Open 2024; 7:e248255. 38656577 10.1001/jamanetworkopen.2024.8255 PMC11043895

[27] von Elm E, Altman DG, Egger M, et al Strengthening the reporting of observational studies in epidemiology (STROBE) statement: guidelines for reporting observational studies. BMJ 2007; 335:806–8. 17947786 10.1136/bmj.39335.541782.AD PMC2034723

[28] Campbell AP, Ogokeh C, Lively JY, et al Vaccine effectiveness against pediatric influenza hospitalizations and emergency visits. Pediatrics 2020; 146:e20201368. 33020249 10.1542/peds.2020-1368

[29] Rha B, Curns AT, Lively JY, et al Respiratory syncytial virus-associated hospitalizations among young children: 2015–2016. Pediatrics 2020; 146:e20193611. 32546583 10.1542/peds.2019-3611 PMC12874392

[30] Uyeki TM, Bernstein HH, Bradley JS, et al Clinical practice guidelines by the Infectious Diseases Society of America: 2018 update on diagnosis, treatment, chemoprophylaxis, and institutional outbreak management of seasonal influenza. Clin Infect Dis 2019; 68:e1–47. 10.1093/cid/ciy866 PMC6653685 30566567

[31] Poehling KA, Edwards KM, Weinberg GA, et al The underrecognized burden of influenza in young children. N Engl J Med 2006; 355:31–40. 16822994 10.1056/NEJMoa054869

[32] Jules A, Grijalva CG, Zhu Y, et al Influenza-related hospitalization and ED visits in children less than 5 years: 2000–2011. Pediatrics 2015; 135:e66–74. 25489015 10.1542/peds.2014-1168 PMC4279064

[33] Poehling KA, Edwards KM, Griffin MR, et al The burden of influenza in young children, 2004–2009. Pediatrics 2013; 131:207–16. 23296444 10.1542/peds.2012-1255 PMC3557405

[34] Centers for Disease Control and Prevention . Influenza antiviral medications: summary for clinicians. Available at: https://www.cdc.gov/flu/professionals/antivirals/summary-clinicians.htm. Accessed 11 July 2024.

[35] Committee on Infectious Diseases . Recommendations for prevention and control of influenza in children, 2023–2024. Pediatrics 2023; 152:e2023063773. 37641879 10.1542/peds.2023-063772

[36] Hamdan L, Probst V, Haddadin Z, et al Influenza clinical testing and oseltamivir treatment in hospitalized children with acute respiratory illness, 2015–2016. Influenza Other Respir Viruses 2022; 16:289–97. 34704375 10.1111/irv.12927 PMC8818823

[37] Roche Pharmaceuticals . Tamiflu (oseltamivir phosphate) [package insert]. US Food and Drug Administration. Available at: accessdata.fda.gov/drugsatfda_docs/label/2012/021087s062lbl.pdf. Revised December 2012. Accessed 28 March 2024.

[38] Rolfes MA, Talbot HK, McLean HQ, et al Household transmission of influenza A viruses in 2021–2022. JAMA 2023; 329:482–9. 36701144 10.1001/jama.2023.0064 PMC9880862

[39] Hatoun J, Correa ET, Vernacchio L. COVID-19 pandemic-related changes in pediatric seasonal respiratory infections. Pediatrics 2022; 150:e2022058618. 35918801 10.1542/peds.2022-058618

[40] Haddadin Z, Schuster JE, Spieker AJ, et al Acute respiratory illnesses in children in the SARS-CoV-2 pandemic: prospective multicenter study. Pediatrics 2021; 148:e2021051462. 33986150 10.1542/peds.2021-051462 PMC8338906

[41] Centers for Disease Control and Prevention . Influenza antiviral medications: summary for clinicians. Available at: cdc.gov/flu/professionals/antivirals/summary-clinicians.htm. Accessed 11 July 2024.

[42] Gao Y, Guyatt G, Uyeki TM, et al Antivirals for treatment of severe influenza: a systematic review and network meta-analysis of randomised controlled trials. Lancet 2024; 404:753–63. 39181595 10.1016/S0140-6736(24)01307-2 PMC11369965

[43] Katzen J, Kohn R, Houk JL, Ison MG. Early oseltamivir after hospital admission is associated with shortened hospitalization: a 5-year analysis of oseltamivir timing and clinical outcomes. Clin Infect Dis 2019; 69:52–8. 30304487 10.1093/cid/ciy860

[44] Louie JK, Yang S, Samuel MC, Uyeki TM, Schechter R. Neuraminidase inhibitors for critically ill children with influenza. Pediatrics 2013; 132:e1539–45. 24276847 10.1542/peds.2013-2149 PMC6637754

[45] Farias JA, Fernández A, Monteverde E, et al Critically ill infants and children with influenza A (H1N1) in pediatric intensive care units in Argentina. Intensive Care Med 2010; 36:1015–22. 20237757 10.1007/s00134-010-1853-1 PMC7095244

[46] Coffin SE, Leckerman K, Keren R, Hall M, Localio R, Zaoutis TE. Oseltamivir shortens hospital stays of critically ill children hospitalized with seasonal influenza: a retrospective cohort study. Pediatr Infect Dis J 2011; 30:962–6. 21997661 10.1097/INF.0b013e318232ede9 PMC3426912

[47] Butler CC, van der Velden AW, Bongard E, et al Oseltamivir plus usual care versus usual care for influenza-like illness in primary care: an open-label, pragmatic, randomised controlled trial. Lancet 2020; 395:42–52. 31839279 10.1016/S0140-6736(19)32982-4

[48] Whitley RJ, Hayden FG, Reisinger KS, et al Oral oseltamivir treatment of influenza in children. Pediatr Infect Dis J 2001; 20:127–33. 11224828 10.1097/00006454-200102000-00002

[49] Fry AM, Goswami D, Nahar K, et al Efficacy of oseltamivir treatment started within 5 days of symptom onset to reduce influenza illness duration and virus shedding in an urban setting in Bangladesh: a randomised placebo-controlled trial. Lancet Infect Dis 2014; 14:109–18. 24268590 10.1016/S1473-3099(13)70267-6

[50] Tenforde MW, Noah KP, O’Halloran AC, et al Timing of influenza antiviral therapy and risk of death in adults hospitalized with influenza-associated pneumonia, FluSurv-NET, 2012–2019. Clin Infect Dis 2025; 80:461–8. 39172994 10.1093/cid/ciae427 PMC11847407

[51] Muthuri SG, Venkatesan S, Myles PR, et al Effectiveness of neuraminidase inhibitors in reducing mortality in patients admitted to hospital with influenza A H1N1pdm09 virus infection: a meta-analysis of individual participant data. Lancet Respir Med 2014; 2:395–404. 24815805 10.1016/S2213-2600(14)70041-4 PMC6637757

[52] Louie JK, Yang S, Acosta M, et al Treatment with neuraminidase inhibitors for critically ill patients with influenza A (H1N1)pdm09. Clin Infect Dis 2012; 55:1198–204. 22843781 10.1093/cid/cis636 PMC12362346

[53] Antoon JW, Hall M, Feinstein JA, et al Guideline-concordant antiviral treatment in children at high risk for influenza complications. Clin Infect Dis 2023; 76:e1040–6. 35867691 10.1093/cid/ciac606 PMC10169402

[54] Antoon JW, Sarker J, Abdelaziz A, et al Trends in outpatient influenza antiviral use among children and adolescents in the United States. Pediatrics 2023; 152:e2023061960. 37953658 10.1542/peds.2023-061960 PMC10681853

[55] Tamiflu Set to Switch to OTC Status . Pharmacy times. Available at: pharmacytimes.com/view/tamiflu-set-to-switch-to-otc-status. Accessed 12 December 2019.

