# Supplementary material for: Antiviral Use Among Children Hospitalized With Laboratory-Confirmed Influenza Illness: A Prospective, Multicenter Surveillance Study
Source: Clin Infect Dis. 2024 Dec 17;81(3):623–33. doi: 10.1093/cid/ciae573 (PMC12497963; doi:10.1093/cid/ciae573)

# SUPPLEMENTARY MATERIAL

**Table S1.** Demographic and clinical characteristics of hospitalized children testing positive for influenza A or B by clinical or research assays, stratified by whether clinical testing was performed, New Vaccine Surveillance Network (12/01/2016–03/31/2020).

| **Characteristic** | **Not clinically tested^1^ (*n*=396)** | **Clinically tested^2^ (*n*=817)** |
| --- | --- | --- |
| Age at presentation (years)—median (IQR) | 3.7 (1.2–7.7) | 3.7 (1.3–8.4) |
| Age group at presentation (years)—*n* (%) |  |  |
| 0–1 | 141 (35.6) | 274 (33.5) |
| 2–4 | 90 (22.7) | 199 (24.4) |
| 5–17 | 165 (41.7) | 344 (42.1) |
| Sex—*n* (%) |  |  |
| Female | 170 (42.9) | 368 (45.0) |
| Male | 226 (57.1) | 449 (55.0) |
| Race and Hispanic origin—*n* (%) |  |  |
| Hispanic | 56/395 (14.2) | 190/810 (23.5) |
| Non-Hispanic Black | 84/395 (21.3) | 250/810 (30.9) |
| Non-Hispanic White | 225/395 (57.0) | 271/810 (33.5) |
| Non-Hispanic other | 30/395 (7.6) | 99/810 (12.2) |
| Insurance status—*n* (%) |  |  |
| Private | 126/388 (32.5) | 222/803 (27.6) |
| Public | 241/388 (62.1) | 525/803 (65.4) |
| Both | 4/388 (1.0) | 16/803 (2.0) |
| Self-pay | 17/388 (4.4) | 40/803 (5.0) |
| Risk factors for influenza complications—*n* (%) |  |  |
| Any risk factor | 343 (86.6) | 730 (89.4) |
| 0–4 years old | 231 (58.3) | 473 (57.9) |
| Respiratory disorder | 118 (29.8) | 244 (29.9) |
| Cardiovascular disorder | 25 (6.3) | 74 (9.1) |
| Neurologic or neuromuscular disorder | 40 (10.1) | 147 (18.0) |
| Hematologic disorder | 16 (4.0) | 75 (9.2) |
| Oncologic or immunocompromising disorder | 10 (2.5) | 46 (5.6) |
| Endocrine disorder | 16 (4.0) | 46 (5.6) |
| Renal or urologic disorder | 7 (1.8) | 19 (2.3) |
| Gastrointestinal or hepatic disorder | 30 (7.6) | 118 (14.4) |
| Genetic or metabolic disorder | 50 (12.6) | 152 (18.6) |
| Signs and symptoms |  |  |
| Days symptomatic at presentation—median (IQR) | 4.0 (2.0–5.0) | 3.0 (2.0–5.0) |
| Day 1 or 2—*n* (%) | 112/394 (28.4) | 327/813 (40.2) |
| Day 3 or after—*n* (%) | 282/394 (71.6) | 486/813 (59.8) |
| Fever—*n* (%) | 372/394 (94.4) | 757/812 (93.2) |
| Cough—*n* (%) | 379 (95.7) | 760 (93.0) |
| Congestion or runny nose—*n* (%) | 346 (87.4) | 715/814 (87.8) |
| Sore throat—*n* (%) | 175/301 (58.1) | 293/604 (48.5) |
| Dyspnea—*n* (%) | 275/393 (70.0) | 536/808 (66.3) |
| Myalgia—*n* (%) | 125/271 (46.1) | 229/554 (41.3) |
| Chills—*n* (%) | 187/349 (53.6) | 380/752 (50.5) |
| Vomiting—*n* (%) | 148 (37.4) | 271/811 (33.4) |
| Diarrhea—*n* (%) | 131/393 (33.3) | 240/812 (29.6) |
| Antiviral use—*n* (%) |  |  |
| Prior use of influenza-specific antivirals | 71/390 (18.2) | 51/814 (6.3) |
| Use of in-hospital antivirals | 133 (33.6) | 519 (63.5) |
| Use of in-hospital antivirals on day 1 or 2 | 122/132 (92.4) | 476/517 (92.1) |
| Received current season influenza vaccine—*n* (%) | 184 (46.5) | 381 (46.6) |
| Research testing—*n* (%) |  |  |
| Not tested for influenza | 0 | 22 (2.7) |
| Tested negative for influenza | 0 | 140 (17.1) |
| Tested positive for influenza | 396 (100.0) | 655 (80.2) |
| Length of stay (days)—median (IQR) | 1.0 (1.0–2.0) | 2.0 (1.0–3.0) |
| ICU admission—*n* (%) |  |  |
| 1 or 2 | 22 (5.6) | 141 (17.3) |
| 3 or after | 2 (0.5) | 11 (1.3) |
| Influenza season—*n* (%) |  |  |
| 2016–2017 | 75 (18.9) | 140 (17.1) |
| 2017–2018 | 93 (23.5) | 203 (24.8) |
| 2018–2019 | 96 (24.2) | 189 (23.1) |
| 2019–2020 | 132 (33.3) | 285 (34.9) |
| Peak influenza season—*n* (%) | 315 (79.5) | 679 (83.1) |
| Study site—*n* (%) |  |  |
| A | 34 (8.6) | 69 (8.4) |
| B | 42 (10.6) | 151 (18.5) |
| C | 42 (10.6) | 93 (11.4) |
| D | 10 (2.5) | 95 (11.6) |
| E | 48 (12.1) | 161 (19.7) |
| F | 33 (8.3) | 67 (8.2) |
| G | 187 (47.2) | 181 (22.2) |

^1^Includes two children who were clinically tested for influenza more than 1 day after antiviral use. ^2^Includes 764 positive and 53 negative results.

**FIGURE S1**. Histogram of days symptomatic at presentation stratified by antiviral receipt.


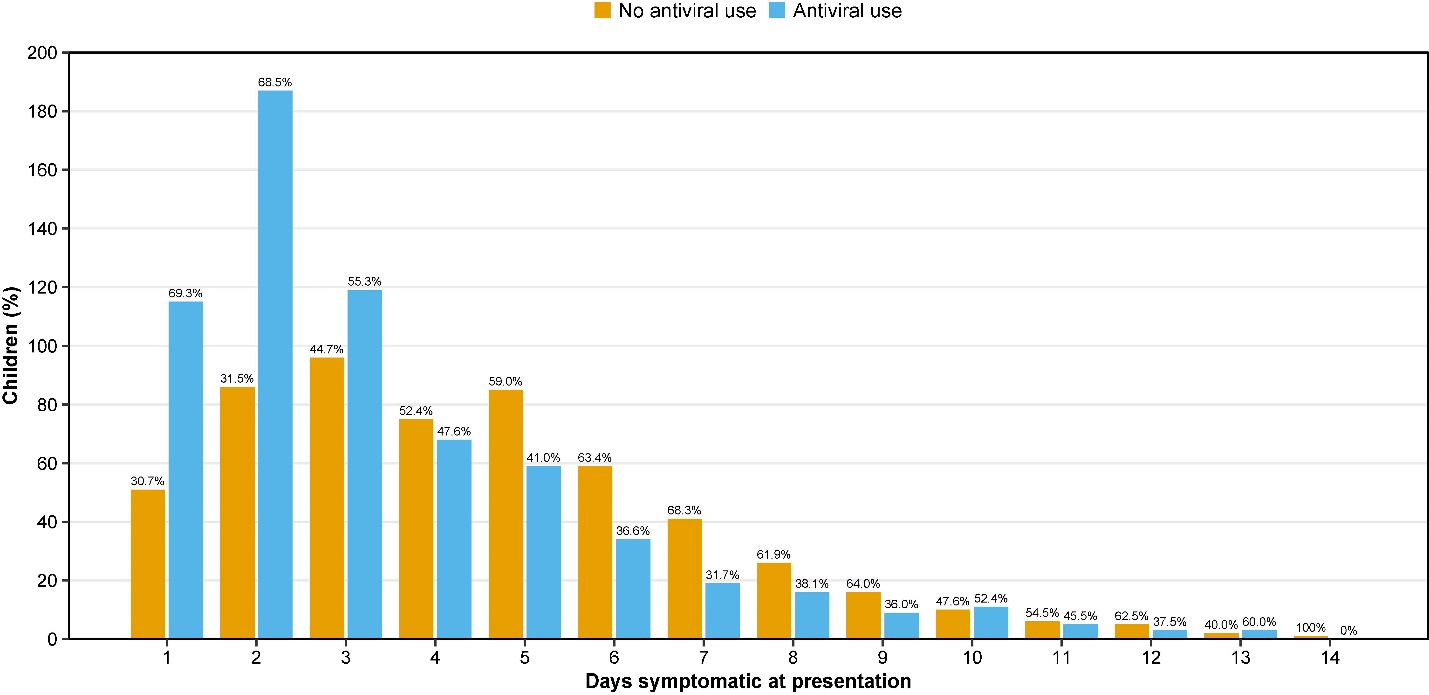


**FIGURE S2**. Factors associated with antiviral receipt among hospitalized children with influenza detected by clinical testing only. Red denotes lower odds of antiviral use and green denotes higher odds of antiviral use.


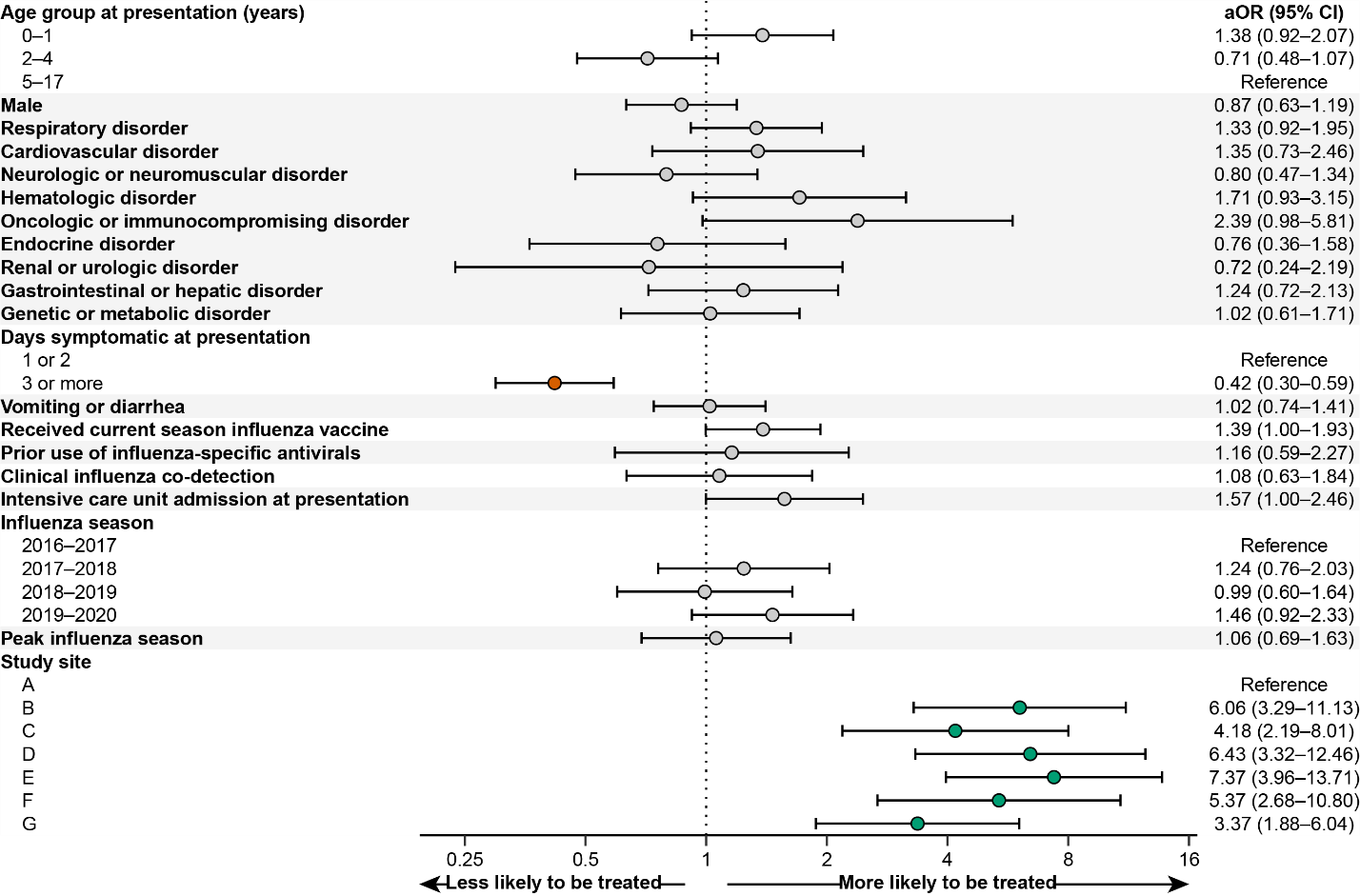

Supplement: ciae573_Supplementary_Data [file ciae573_supplementary_data.docx]
